# Spectral dependence of lipofuscin fluorescence lifetimes revealed by FLIM with a superconducting nanowire single-photon detector

**DOI:** 10.1117/1.JBO.31.6.066501

**Published:** 2026-06-24

**Authors:** Pavel Morozov, Vladislav Andreev, Marina Yakovleva, Alex Kostyukov, Tatiana Feldman, Anastasia Ryabova, Igor Romanishkin, Marina Shirmanova, Gregory Goltsman, Vladimir Kuzmin, Mikhail Ostrovsky, Alexandra Arkhipchenko, Eugene Maksimov, Wolfgang Becker, Vladislav Shcheslavskiy

**Affiliations:** a Moscow Pedagogical State University, Moscow, Russia; b National University Higher School of Economics, Moscow, Russia; c N.M. Emanuel Institute of Biochemical Physics RAS, Moscow, Russia; d Lomonosov Moscow State University, Moscow, Russia; e Russian Academy of Sciences, Prokhorov General Physics Institute, Moscow, Russia; f Privolzhsky Research Medical University, Novgorod, Russia; g Becker & Hickl GmBH, Berlin, Germany

**Keywords:** lipofuscin, fluorescence lifetime imaging microscopy, FLIM, A2E, bisretinoids, retinal pigment epithelium, multimode fiber coupled large active area SNSPD, decay curves, time-correlated single-photon counting

## Abstract

**Significance:**

The noninvasive assessment of oxidative stress in the retinal pigment epithelium (RPE), a key factor in the pathogenesis of age-related macular degeneration (AMD), is an important aspect in ophthalmic diagnostics. Although fundus autofluorescence (FAF) is clinically used, its diagnostic power is limited. Fluorescence lifetime imaging ophthalmoscopy shows promise, but the photophysical underpinnings of lifetime changes in lipofuscin—the dominant RPE fluorophore—remain poorly understood. Lipofuscin granules (LGs) in the RPE are a primary source of fundus autofluorescence and are critically involved in the pathogenesis of AMD. This study characterizes the spectral and lifetime properties of LGs using a fluorescence lifetime imaging microscopy (FLIM) system integrated with a wide-range sensitivity, large-active-area superconducting nanowire single-photon detector (SNSPD) coupled with a standard 50  μm multimode fiber.

**Aim:**

We present experiments on multispectral fluorescence lifetime imaging with a detailed analysis of the fluorescence decays and spectral profiles of LGs in the RPE cells to probe the molecular oxidative state of RPE lipofuscin, aiming to improve AMD understanding and propose a method for early diagnosis of retinal pathologies.

**Approach:**

We use a cutting-edge FLIM system integrated via a multimode fiber with a large-active-area SNSPD. Unlike previous studies, our approach provides component-resolved and spatially resolved fluorescence decay kinetics across a broad spectral range (500 to 1000 nm), which is typically inaccessible to conventional detectors. LGs were isolated from 100 human (aged 50 to 75 years) donor RPE cells and subjected to *in vitro* photo-oxidation to simulate AMD-like conditions. Steady-state spectroscopy revealed a blue shift in emission following photo-oxidation.

**Results:**

FLIM analysis, employing a tri-exponential decay model, showed a significant increase in the mean fluorescence lifetime $\tau_{m}$ from ∼247 to ∼587  ps after photo-oxidation. Crucially, we identified a pronounced direct spectral dependence of fluorescence lifetimes $\tau_{m}$ versus wavelength: for native LGs, $\tau_{m}$ increased from 136 to 319 ps at registration range 500 to 650 nm; for photo-oxidized LGs, it increased from 174 to 623 ps over the same region. High-performance liquid chromatography confirmed the degradation of the bisretinoid A2E and accumulation of oxidized derivatives, correlating with the observed photophysical changes.

**Conclusions:**

These findings demonstrate that component-resolved visible-near infrared spectral FLIM provides a sensitive, noninvasive approach to probe the molecular oxidative state of RPE lipofuscin, offering potential for early diagnosis of retinal pathologies.

## 1 Introduction

Lipofuscin granules (LGs) are autofluorescent pigments^1^ of lipid and glycoprotein origin that play a critical role in cellular aging and neurodegenerative diseases.^2^ These granules accumulate within the lysosomal compartment of postmitotic cells throughout the body,^3^ including myocardium,^4^ retina, and ganglion cells,^5^ inside the human brain,^6^ neurons,^2^ and various visceral organs such as the liver, kidneys, and adrenal glands.^7^ Although LGs volume increases progressively over time, their intracellular concentration is considered a primary biomarker of aging.^8^^,^^9^

In the eye, LGs originate in the retinal pigment epithelium (RPE) from the incomplete lysosomal degradation of photoreceptor outer segment debris.^10^ These granules are the principal source of fundus autofluorescence (FAF).^11^ Chemically, LGs comprise over two dozen distinct fluorophores, primarily bis-retinoids and their photo-oxidation products,^12^ with N-retinylidene-N-retinyl-ethanolamine (A2E) being the most extensively characterized.^13^ The excessive accumulation of LGs in the RPE is a hallmark of aging and is strongly associated with the pathogenesis of age-related macular degeneration (AMD) and other retinal diseases.

AMD is a leading cause of irreversible vision loss in individuals over the age of 50. The pathogenesis of AMD involves vascular dysfunction and metabolic impairment of the central retina (macula), which is critical for central vision.^14^^,^^15^ Consequently, there is an urgent need for advanced non-invasive diagnostic tools. Although fundus autofluorescence (FAF) is currently employed for clinical imaging,^16^^,^^17^ its diagnostic capabilities remain limited when based solely on fluorescence intensity and spectral distribution.

To achieve a more comprehensive characterization of retinal fluorophores, fluorescence lifetime imaging microscopy (FLIM) has been increasingly adopted. FLIM provides spatial resolution of fluorescence decay kinetics, offering insights that intensity-based imaging cannot capture.^18^ Previous FLIM-based studies^19^^,^^20^ have demonstrated that the fluorescence lifetimes of LGs increase during the progression of retinal pathologies, although the underlying mechanisms remain poorly understood. Comparative studies of RPE LGs in healthy and pathological states, along with an analysis of their chloroform extracts, suggest that prolonged fluorescence lifetimes are characteristic of oxidized bisretinoids.^21^^,^^22^ These findings indicate that FLIM can effectively identify early biomarkers of AMD.

Furthermore, the burgeoning interest in near-infrared (NIR) FLIM^23^ underscores the necessity for enhanced detection sensitivity. In this study, we employed a large-active-area superconducting nanowire single-photon detector (SNSPD/SSPD), which significantly extends the capabilities of FLIM for biomedical research across both the visible and NIR spectral ranges.^24^^,^^25^

Unlike previous studies that relied on bulk measurements of LG suspensions to obtain sample-averaged data, our FLIM-SNSPD approach enables the visualization of the spatial distribution of LGs within their microenvironment. To further elucidate the mechanisms of retinal aging, we characterized the spectral dependence of fluorescence decay kinetics and generated lifetime distribution histograms using a tri-exponential model.^26^^–^^28^ This analysis clearly illustrates the structural and chemical transitions induced by photo-oxidation. The comprehensive data provided by these methods offer deeper insights into the oxidative processes associated with retinal aging, potentially facilitating the early-stage diagnosis of AMD and other ocular pathologies.

## 2 Materials and Methods

### 2.1 Sample Preparation

Lipofuscin granules (LGs) were isolated from the retinal pigment epithelium (RPE) cells of 100 donor human cadaver eyes. The resulting cellular material was homogenized, and LGs were subsequently purified from the pooled homogenate via standard extraction and previously established protocol^29^ and suspended in 0.1 M potassium phosphate buffer (pH 7.3). The donor material was provided by the Eye Tissue Bank of the S. Fyodorov Eye Microsurgery Federal State Institution, sourced from the Moscow Forensic Medical Examination Bureau.^30^ Criteria for inclusion were donors aged 50 to 75 years with no history of ocular pathology.

Eyes were processed within 10 h postmortem following corneal recovery for transplantation. After the removal of the lens, vitreous body, and retina, the ocular fundus was carefully inspected. To prevent photo damage, all procedures were conducted under dim light. The concentration of LGs was determined using a hemocytometer, with an initial concentration of $3\times 10^{9}\text{ granules}/\mathrm{ml}$.

Photo-oxidation of LGs samples was performed using a 395 nm LED for 20 min at an irradiance of $3\;W/{\mathrm{cm}}^{2}$, a dosage sufficient to induce comprehensive photo-oxidation of LGs bisretinoids.

### 2.2 High-Performance Liquid Chromatography (HPLC)

To validate the results obtained from VIS-NIR spectroscopy and FLIM, synthetic N-retinylidene-N-retinyl-ethanolamine (A2E) was employed as a reference standard. A2E was synthesized by reacting all-trans-retinal and ethanolamine in a mixture of acetic acid and ethanol following the established protocol.^31^ The identity of the synthesized A2E was confirmed using a 7T LTQ FT mass spectrometer (Thermo Electron Corporation, United States) equipped with an electrospray ionization (ESI) source;^32^ data were processed with Qual Browser 1.4 software. The purity of A2E and the separation of bisretinoids from their photo-oxidation products in RPE-derived LGs chloroform extracts were assessed by HPLC (Knauer system, Germany; Kromasil-100-5-C18 column, 4×250  mm, 5  μm). A linear gradient from 80% acetonitrile/20% water (0.05% TFA) to 100% acetonitrile over 20 min (1.0  mL/min flow rate) achieved separation, with detection by a K-2501 photometric detector.^32^

Measurement accuracy was determined based on three independent chromatographic runs for each sample. The relative content of each component was calculated as a percentage of the total integrated peak area. Because the majority of the detected photo-oxidation products remain unidentified, their relative concentrations were estimated assuming uniform extinction coefficients. Statistical significance between groups was assessed using Student’s t-test, with p-values <0.05 considered statistically significant.

### 2.3 VIS-NIR Spectroscopy and Fluorescence Lifetime Imaging Microscopy (FLIM)

Fluorescence VIS-NIR spectroscopy and FLIM data measurements were performed using a custom-built confocal galvano-scanner system based on DCS-120 (Becker & Hickl GmbH, Germany) coupled to a Zeiss Axio Observer A1 microscope equipped with 40×/0.8 and 60×/1.49 NA objectives and integrated with a superconducting nanowire single-photon detector. Samples were excited using a 50 ps diode laser operating at 473 nm with a repetition rate of 50 MHz, and an average power of $50\;\mu W/{\mathrm{cm}}^{2}$ BDL-472-SMC (Becker & Hickl GmbH, Germany). Fluorescence spectra were recorded from 500 to 1000 nm with a 10 nm step using a monochromator with a grating of 1200  lines/mm (M140 Photonics Instruments, Belarus) connected to the SNSPD via 50  μm core-diameter multimode fiber ClearCurve OM2 (Corning, United States). FLIM data were acquired using the SPC-150NX time-correlated single-photon counting (TCSPC) board (Becker & Hickl GmbH, Germany).

For comparative validation and showing the same tendency, spectroscopy measurements were also performed on a Zeiss LSM-710-NLO laser-scanning microscope; the Plan-Apochromat 20×/0.8 NA objective (Zeiss, Germany) was used. The spectra were recorded under two-photon excitation at $\lambda_{\mathrm{exc}}=800\;\mathrm{nm}$ with a Chameleon Ultra II (Coherent, USA) with pulse width 140 fs, repetition rate 80 MHz, and average power $10\;\mathrm{mW}/{\mathrm{cm}}^{2}$. This system was equipped with an SPC-150 time-correlated single-photon counting module and a GaAsP HPM-100-07 hybrid detector (Becker & Hickl GmbH, Germany).

A superconducting nanowire single-photon detector (OPRS-SW-70-MMF, Scontel) significantly outperforms conventional detectors in terms of quantum efficiency, dark count rate, dead time, and timing jitter, making them an ideal solution for advanced biomedical applications in both the visible and NIR spectral ranges.^33^ The detector, featuring a $50\times 50\;\mu {\text{m}}^{2}$ active area,^34^ was coupled to 50  μm core-diameter multimode fiber ClearCurve OM2 (Corning, United States) and housed in a vacuum cryostat (based on compact Gifford-McMahon closed-cycle refrigeration) operating at 2.3 K. The SNSPD was connected to the scanner via the same type 50  μm multimode fiber. The system detection efficiency exceeded 60% across a broad spectral range of 500 to 1000 nm. The measured instrument response function of the entire system was 108 ps at 473 nm.^35^

For measurements, the aqueous suspension of LGs was placed in a FluoroDish (FD35, World Precision Instruments, United States). The laser excitation beam was directed via two galvanometric mirrors for pixel-by-pixel scanning of the sample.^28^ The resulting fluorescence signal was collected through the same scanning optics, separated from the excitation light by a dichroic mirror, and focused into the multimode fiber coupler of the SNSPD. Image acquisition and lifetime analysis were performed using SPCImage software (Becker & Hickl, Germany).

### 2.4 Triple-Exponential Decay Function Modeling and Convergence

To quantify the contribution of individual fluorophores components, the recorded decay curves were modeled using a multiexponential function^26^^–^^28^ accounting for the instrument response function (IRF):

$$I(t)=\int_{0}^{t}F(t)\cdot R(t)dt,$$

where R(t) is an instrument response function and F(t) is a model function:

$$F(t)=\Sigma (A_{j}\cdot e^{\left(\frac{-t}{\tau_{j}}\right)}).$$

The mean fluorescence lifetime $\tau_{m}$ was calculated using the formula:


$$\tau_{m}=\Sigma (A_{j}\cdot \tau_{j})/\Sigma A_{j}.$$

Throughout the fitting procedure of the fluorescence decay curves, the $\chi^{2}$ demonstrated convergence within the expected interval of 0.95 to 1.05 and indicating a good fitting during the modeling.

## 3 Results and Discussion

### 3.1 Fluorescence Spectra of Lipofuscin Granules

**Fig. 1:**
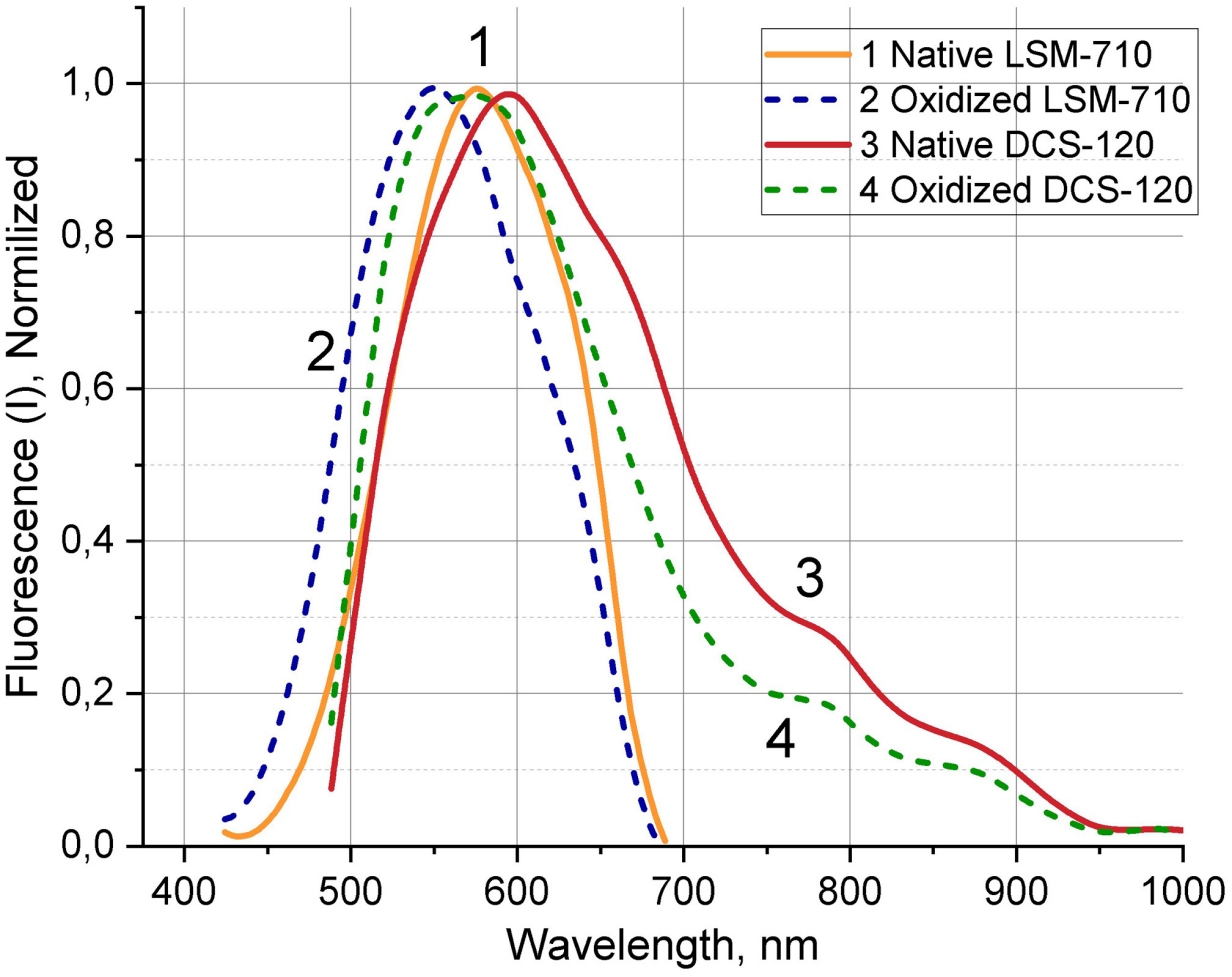
Fluorescence spectra of lipofuscin granules before (solid line) and after photo-oxidation (dash line). (1,2) Acquired on the LSM-710-NLO system, two-photon excitation $\lambda_{\mathrm{exc}}=800\;\mathrm{nm}$. (3,4) Acquired on the DCS-120 FLIM-SNSPD system at $\lambda_{\mathrm{exc}}=473\;\mathrm{nm}$. All spectra were smoothed using the SavitzkyGolay fit.

The resulting fluorescence spectra are presented in Fig. 1, photo-oxidation significantly modifies the fluorescence intensity in both the 500 to 600 nm and 600 to 700 nm spectral ranges, resulting in a pronounced hypsochromic blue shift of the emission maximum. This spectral transformation is attributed to the degradation of long-wavelength fluorophores, such as A2E, and the simultaneous accumulation of their oxidized derivatives which exhibit shorter-wavelength fluorescence.^21^^,^^36^ These spectral changes directly indicate an increased concentration of oxy-bisretinoids within the photo-oxidized LGs.

It is important to note that the spectra acquired on the LSM-710-NLO system (curves 1, 2; two-photon excitation at 800 nm) and on the DCS-120 FLIM-SNSPD system (curves 3, 4; one-photon excitation at 473 nm) are not directly quantitatively comparable. Two-photon and one-photon excitations follow different quantum mechanical selection rules and excite distinct subsets of fluorophores due to wavelength-dependent absorption cross-sections. Therefore, the absolute fluorescence intensities and the exact positions of the emission maxima should not be compared directly between the two systems. Instead, the data are presented for two separate purposes: (i) to demonstrate the superior near-infrared sensitivity of the SNSPD compared with a conventional GaAsP detector, and (ii) to show that the blue shift of the emission maximum upon photo-oxidation is qualitatively reproducible under both excitation modalities, confirming the robustness of this spectral transformation. The small (∼25  nm) red shift observed for one-photon excitation (473 nm) relative to two-photon excitation (800 nm) likely reflects the preferential excitation of longer-wavelength-absorbing fluorophores at 473 nm, which are not as efficiently excited at 800 nm under two-photon conditions.

### 3.2 FLIM of Lipofuscin: before and after Photo-Oxidation

**Fig. 2:**
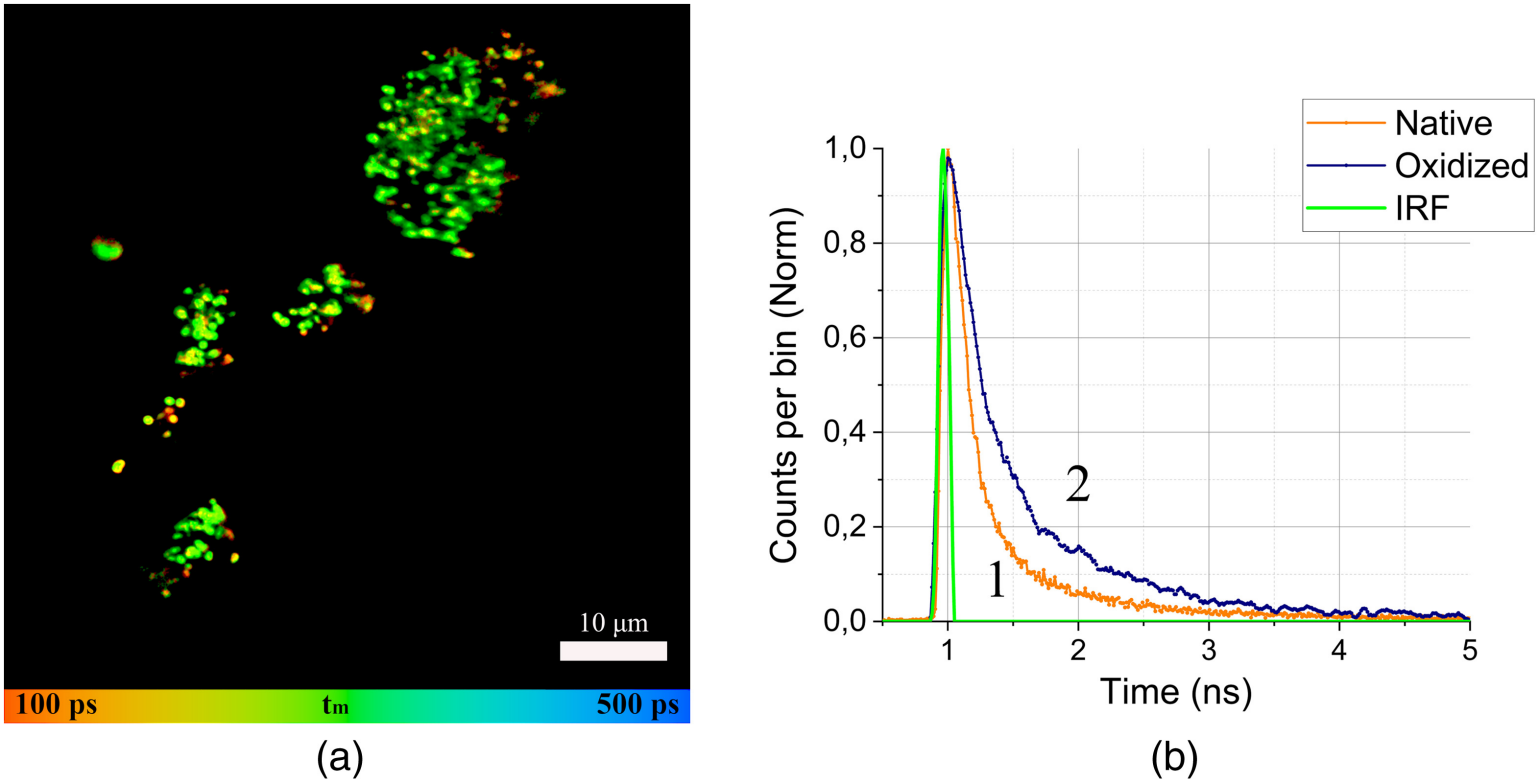
(a) FLIM of native lipofuscin granule clusters for $\tau_{m}$, pseudocolor represents the mean fluorescence lifetime, image size: 512×512  pixels. (b) Normalized mean fluorescence decay curves $\tau_{m}$ of lipofuscin obtained from FLIM: native (1) and photooxidized (2).

Figure 2 presents a FLIM image of the native sample, where individual spherical lipofuscin granules are clearly resolved. The lack of grain merging and high image fidelity demonstrate the superior spatial resolution and contrast provided by the combined FLIM-SNSPD system. Fluorescence images were collected for 60 s to avoid deep photo-oxidation of LGs by irradiation of the 473 nm laser.

The fluorescence decay curves [Fig. 2(b)] obtained from FLIM were analyzed with a tri-exponential model,^26^^–^^28^ $\chi^{2}$ value was 0.98. The total number of photons per decay curve was at least 7000.

The fluorescence decay parameters for LGs before and after photo-oxidation ($\lambda_{\mathrm{exc}}=473\;\mathrm{nm}$, $\lambda_{\mathrm{reg}}>500\;\mathrm{nm}$) are summarized in Table 1. Following irradiation, a significant increase in $\tau_{m}$ was observed, indicating a population shift of fluorophores toward oxidized states. Multiexponential analysis confirms this shift, showing an increased relative contribution from oxidized fluorophores alongside a concurrent decrease from their nonoxidized precursors.

This redistribution is quantified by the amplitude ratio $(A_{2}+A_{3})/A_{1}$, which more than doubled after photo-oxidation. Because the $\tau_{1}$ component is assigned to oxidized bisretinoid species^37^ (characterized by longer lifetimes). This result is consistent with the overall elongation of the fluorescence decay and confirms that photo-oxidation significantly enriches LGs with oxidized bisretinoid derivatives.

**Table 1:** Fluorescence lifetimes $\tau_{j}$ components and their amplitudes – $A_{j}$ (%) contribution to the total fluorescence.

LG sample	$\tau_{1}$, ps	$A_{1}$, %	$\tau_{2}$, ps	$A_{2}$, %	$\tau_{3}$, ps	$A_{3}$, %	$\tau_{m}$, ps	$(A_{2}+A_{3})/A_{1}$
1. Native	168	84.5	672	10.5	2051	5	247	0.18
2. Photooxidized	202	68.5	731	20.5	2714	11	587	0.46

**Fig. 3:**
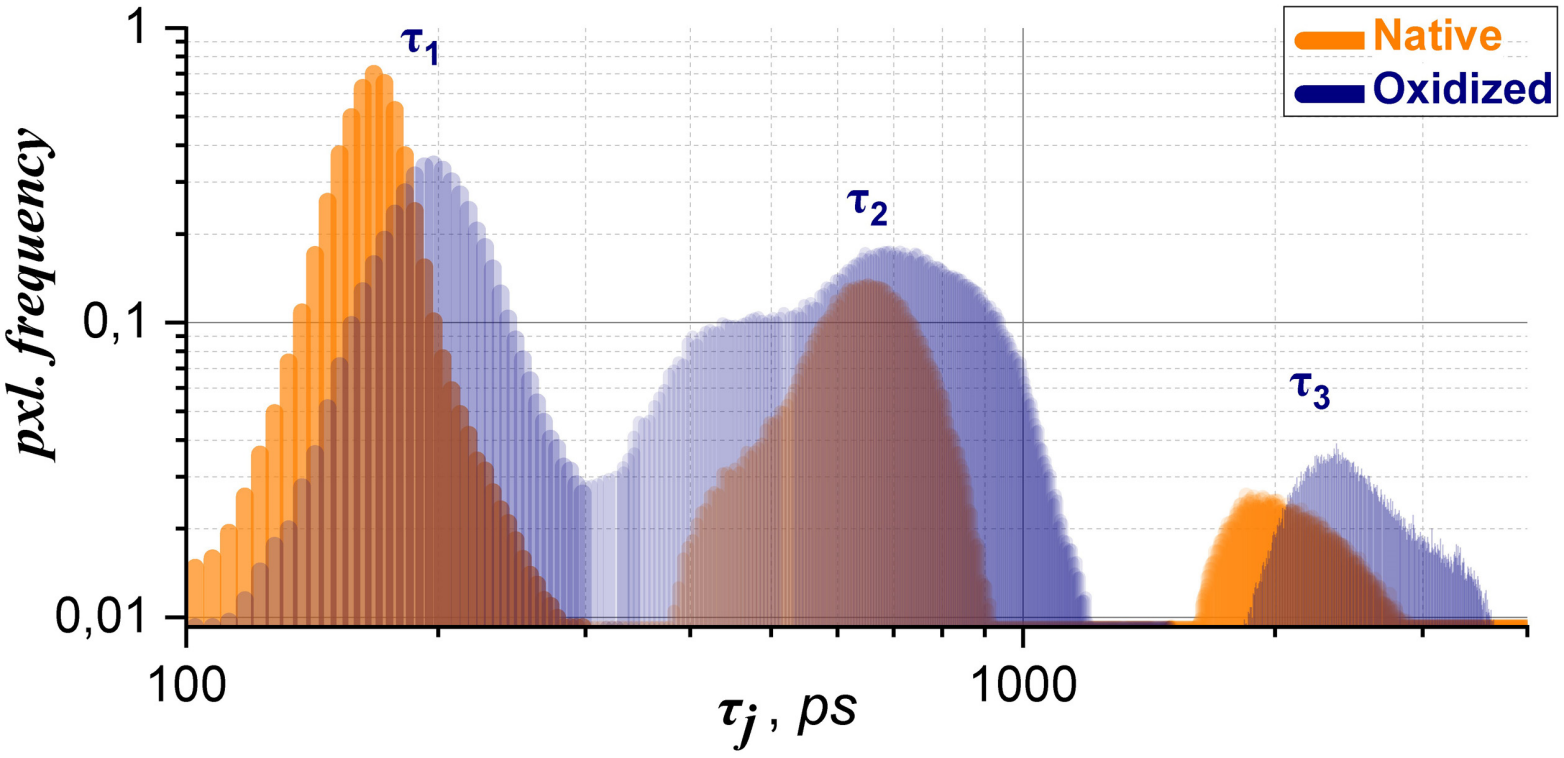
Logarithmic pixels frequency versus lifetime distribution histograms for all the lifetime components $\tau_{j}$, native (orange) and after photooxidation (navy).

Figure 3 presents logarithmic pixel frequency versus lifetime distribution histograms derived by SPCImage from the multiexponential decomposition of the fluorescence decay kinetics for each $\tau_{j}$ component, comparing native and photo-irradiated LGs. Following 395 nm irradiation, a distinct shift in the distributions was observed, indicating altered fluorophores microenvironments and oxidation states. The profiles show significant broadening and redistribution across the short-lived (100 to 300 ps), middle-lived (300 to 1500 ps), and long-lived (1500 to 4000 ps) regions. For the $\tau_{1}$ component, photo-oxidation induced time broadening and a shift in the peak value from 168 to 202 ps, reflecting a relative decrease in the fastest decay contributions. A similar trend was observed for $\tau_{2}$ and $\tau_{3}$ (Tables 1 and 2).

**Fig. 4:**
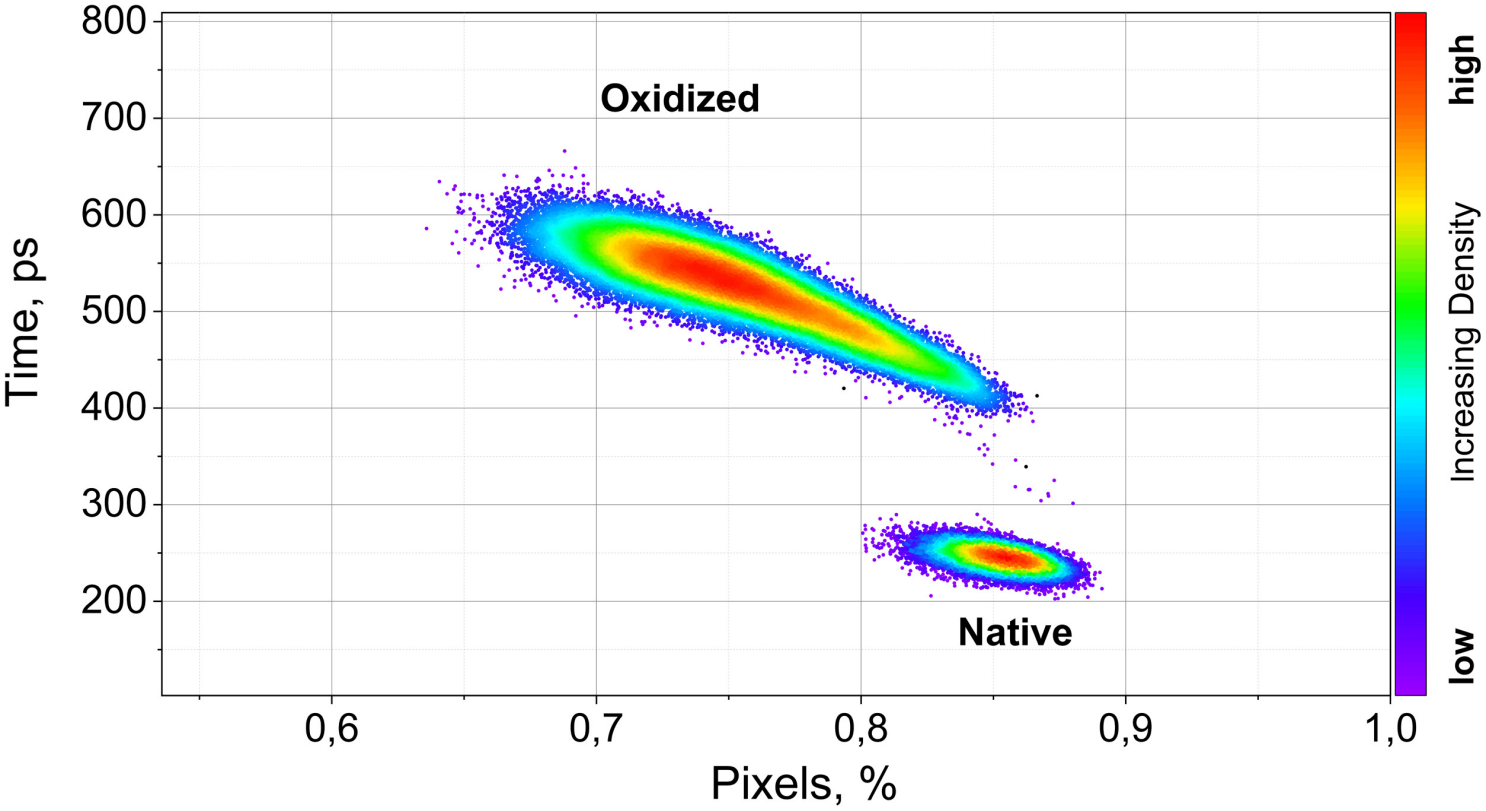
Lifetime distribution for mean component $\tau_{m}$: native and photo-oxidized LGs.

Figure 4 shows lifetimes distribution for $\tau_{m}$. One can observe that the distribution has changed after photo-oxidation. Photo-oxidation induces a shift toward long-lived emissive states across the mean fluorescence lifetime $\tau_{m}$_._ Interestingly, not only $\tau_{m}$ has increased upon oxidation, but also the dispersion of a distribution has increased.

The quantitative analysis of these distributions is summarized in Tables 1 and 2. The data confirm a significant increase in the fluorescence lifetimes for all three individual components ($\tau_{j}$), as well as a corresponding rise in the mean fluorescence lifetime ($\tau_{m}$) for photooxidized LGs.

**Table 2:** Distribution of lifetime components $\tau_{1}$, $\tau_{2}$, $\tau_{3}$, $\tau_{m}$ (Fig. 3) for $I_{\text{norm}}>1\%$.

Sample	$\tau_{1}$, ps	$\tau_{2}$, ps	$\tau_{3}$, ps	$\tau_{m}$, ps
1. Native	114 to 287	302 to 913	1596 to 2681	247
2. Photooxidized	151 to 395	283 to 1801	1839 to 3712	587

Photo-oxidation promotes the prevalence of long-lived emissive states across all decay components ($\tau_{j}$), resulting in a significant increase in the mean lifetime ($\tau_{m}$) from 247 to 587 ps. The methodological novelty of this study lies in the component-resolved pixels frequency analysis of fluorescence decay kinetics. By extracting the pixels frequency versus lifetime distribution histograms for each individual $\tau_{j}$ from originally measured $\tau_{m}$, we could quantify the specific contributions of distinct lifetime populations to the total emission.

**Fig. 5:**
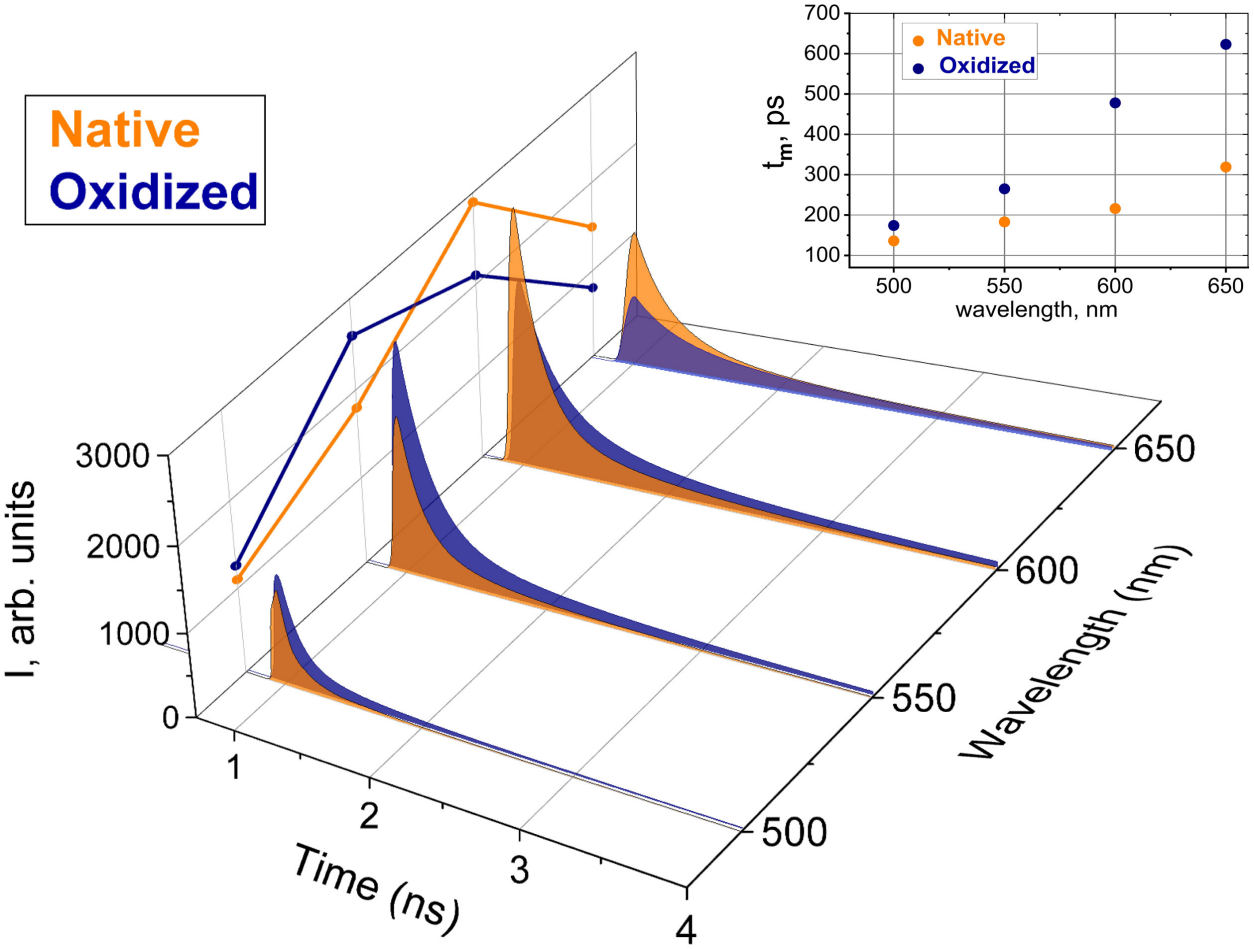
Spectral dependence of fluorescence decay curves of lipofuscin granules for $\tau_{m}$ : orange-native, navy-photooxidized. Insert: Graph of spectral dependence for $\tau_{m}$ versus wavelength.

Fluorescence decay curves were recorded across an emission range of 500 to 650 nm (50 nm step and 1 nm bandwidth of monochromator) under 473 nm excitation. Our findings reveal a direct wavelength dependence of the fluorescence lifetimes for this region (Fig. 5). Specifically, as the detection wavelength increased from 500 to 650 nm, $\tau_{m}$ increased from 136 to 319 ps for nonoxidized LGs and from 174 to 623 ps for photo-oxidized samples.

A similar result was previously reported in Ref. 37, but only for native LGs and under different excitation conditions. Our results extend these observations by demonstrating that while photo-oxidation shifts the absolute lifetime values, the characteristic direct spectral dependence remains a fundamental property of lipofuscin fluorescence (Table 3).

**Table 3:** Spectral dependence of fluorescence lifetimes in LGs.

$\text{Sample}\backslash \lambda_{\mathrm{reg}}$ (nm)	500	550	600	650
1. Native $\tau_{m}$ (ps)	136	183	216	319
2. Photooxidized $\tau_{m}$ (ps)	174	265	478	623

Consequently, shorter-lived emissive states predominate in the short-wavelength region, while longer-lived components are more prevalent at longer wavelengths. This indicates that the spectral blue shift of the fluorescence (Fig. 1) is associated with a redistribution between short-lived and long-lived fluorophores.

Analysis of the steady-state fluorescence spectra (Fig. 1) and wavelength-resolved kinetics (Fig. 5) shows that the postirradiation fluorescence enhancement between 500 and 600 nm stems from a larger population of long-lived emitters; this indicates a greater abundance of photo-oxidized bisretinoids within the lipofuscin granules (LGs). Conversely, the intensity decreases in the 600 to 800 nm region for oxidized LGs, points to a depletion of the original short-lived fluorophores (Table 1). Together, these results demonstrate that oxidation systematically redistributes the fluorophores population, favoring longer-lived, blue-shifted derivatives.

### 3.3 High-Performance Liquid Chromatography (HPLC)

To confirm the photo-oxidation process of bisretinoids in lipofuscin granules, HPLC analysis was performed. The relative bisretinoid content (see methods) data are presented in Table 4. All peaks were divided into groups for ease of data presentation: highly oxidized bisretinoidsoxy-bisretinoids (1), weakly oxidized A2Eoxy-A2E (2), A2E (3), and other bisretinoids - bisretinoids (4).

**Table 4:** Relative content of BisRets and BisRets-OX in the RPE LGs before and after exposure to accelerated protons or visible light. Data were obtained as relative abundances from chromatographic data with detection at 430 nm. Chloroform extract samples from the RPE LGs. oxy-bisretinoids (1), oxy-A2E (2), A2E (3), and bisretinoids (4).

Peak/group	Control	Light 5 min	Light 10 min
1. Oxy-bisretinoids	32.3±0.7	35.1±0.6	37.3±0.5
2. Oxy-A2E	2.7±0.3	4.1±0.4	5.3±0.2
3. A2E	34.7±0.6	31.2±0.5	28.3±0.5
4. Bisretinoids	30.3±0.5	29.6±0.7	29.1±0.6

Data are shown as mean ± SD from three independent HPLC experiments conducted for each type of radiation, p<0.05.

Chromatographic analysis showed that LGs photo-oxidation substantially diminished the A2E and isomer peak (peak 3 and 4) while simultaneously increasing the content of multiple peaks corresponding to oxidized derivatives (peak 1 and 2).^21^ These results align perfectly with the spectral FLIM data, confirming that the blue-shifted fluorescence maximum of oxidized LGs results from the accumulation of these photo-oxidized products.

## 4 Conclusions

This study demonstrated the high efficiency of FLIM integrated with a large-active-area 50×50  μm SNSPD coupled with a multimode fiber for the precise characterization of retinal pigment epithelial lipofuscin. By utilizing the enhanced sensitivity of SNSPDs in the VIS-NIR range, we successfully resolved the complex decay kinetics of lipofuscin granules (LGs) across a broad spectral interval (500 to 1000 nm).

LGs photo-oxidation, simulating AMD-like conditions, leads to a significant prolongation of the mean fluorescence lifetime ($\tau_{m}$), rising from ∼247 to ∼587  ps in the range from 500 to 1000 nm. This shift is driven by a redistribution of lifetime populations, specifically a decrease in short-lived components and the emergence of long-lived oxidized states.

A direct wavelength dependence of fluorescence lifetimes in RPE lipofuscin was identified. We observed that $\tau_{m}$ increases as the emission wavelength increases, a trend that persists in both native and photo-oxidized states. The observed blue shift in the fluorescence spectrum originates from a redistribution of population between short- and long-lived fluorophores.

HPLC analysis confirmed that these lifetime shifts correlate with the degradation of A2E and the accumulation of oxidized bisretinoids, validating the use of FLIM as a surrogate marker for molecular changes in the RPE and retina.

Our results expand the analytical capabilities of autofluorescence imaging by transitioning from simple intensity-based measurements to component-resolved spectral FLIM. This approach offers a robust framework for the early noninvasive diagnosis of AMD and other age-related retinal pathologies, providing deeper insights into the metabolic state of the retinal pigment epithelium.

## Data Availability

Data underlying the results presented in this paper are not publicly.
